# Revision Labiaplasty After Penile Inversion Vaginoplasty Using Costal Cartilage Allograft

**DOI:** 10.5152/tud.2022.22079

**Published:** 2022-11-01

**Authors:** Nicholas Sellke, Michael Callegari, Kirtishri Mishra, Tobias Long, Shubham Gupta

**Affiliations:** 1 University Hospitals Cleveland Medical Center – Urology Institute, Cleveland, OH, USA

**Keywords:** Labiaplasty, vaginoplasty, gender affirmation, cartilage, allograft, reconstruction

## Abstract

**Background:**

Gender-affirming surgery leads to high satisfaction for patients; however, patients often require revision surgery. Revision labiaplasty is one of the most common surgeries following vaginoplasty. The labia minora commonly become incorporated into the labia majora and lose definition even after revision leading to patient dissatisfaction.

**Description of Technique:**

We propose a technique of incorporating cadaveric costal cartilage allograft into the revised labia minora to increase the definition.

**Patient and Methods:**

The procedure was demonstrated in a 38-year-old MTF patient who had previously undergone inversion vaginoplasty. The cartilage allograft was incorporated within the labia minora flaps during labiaplasty.

**Results:**

The patient had lasting definition of her labia minora post-operatively at 6 weeks without adverse effects.

**Conclusion:**

We believe that this technique may be effective and safe in patients requesting more defined labia minora after vaginoplasty.

**Main Points**

- Poorly defined labia minora is a frequent issue bothering patients after gender-affirming vaginoplasty.
- Revision labiaplasty necessitates a novel solution as traditional techniques often lose definition post-operatively.
- The use of costal cartilage within the labia minora flap can add durable definition.

## Background

Gender-affirming surgery (GAS) leads to high levels of patient satisfaction as well as psychosocial relief for transgender patients suffering from gender dysphoria.^1-3^ Gender-affirming surgery for transgender women includes a multitude of procedures altering the patient’s entire physical body, namely the chest and genitals to align their body with their identified gender. The most common genital affirming procedure is the penile inversion vaginoplasty, which uses the penile and scrotal skin to create a functional vaginal canal and clitoris in addition to labia majora and minora.^2-4^

The penile inversion vaginoplasty procedure has led to significant patient satisfaction with regards to aesthetic outcome; however, despite this many patients often undergo revision; between 30% and 90% of which include a revision labiaplasty.^5,6^ Most commonly, patients express a desire for increased definition of the labia minora.^7,8^ Specifically, patients express displeasure with post-reconstructive, ill-defined, and flat-appearing labia minora that become part of the more prominent labia majora.^9^

There is limited literature regarding feminizing labiaplasty in transgender patients. Literature describing penile inversion vaginoplasty briefly outlines the initial formation of labia minora from bilateral flaps of penoscrotal tissue (formerly along the raphe) that are embedded to subcutaneous fat alongside the introitus.^9^ Post-operatively, the definition and presence of these labia minora can often become diminished prompting desire for revision. Techniques for revision have been proposed but even the authors of some techniques admit they are not satisfactory.^10^

To achieve durable and cosmetically appealing labia minora, we propose a new technique for labial reconstruction using fresh frozen costal cartilage allograft (MTF Biologics, Edison NJ). Sourced from donor human costal cartilage, this material is primarily utilized in revision rhinoplasty.^11,12^ Frozen costal cartilage allografts have gained popularity among plastic and reconstructive surgeons due to their ease of use, customizable nature, structural properties, and above all, the elimination of additional procedures, scars, and pain from an autologous source.^13^ We hypothesized that costal cartilage could similarly demonstrate an effective means to perform primary or revision labiaplasty and ultimately improve cosmetic and functional outcomes.

## Description of the Technique

Our novel technique begins with lifting thin labia majora flaps. Then, to prevent later loss of definition, we place a thin section of costal cartilage allograft within the flap before suturing the flap in place. The addition of cartilage adds additional, lasting definition to the labia minora in patients with a lack of definition after vaginoplasty.

## Patient and Methods

**Figure 1.**
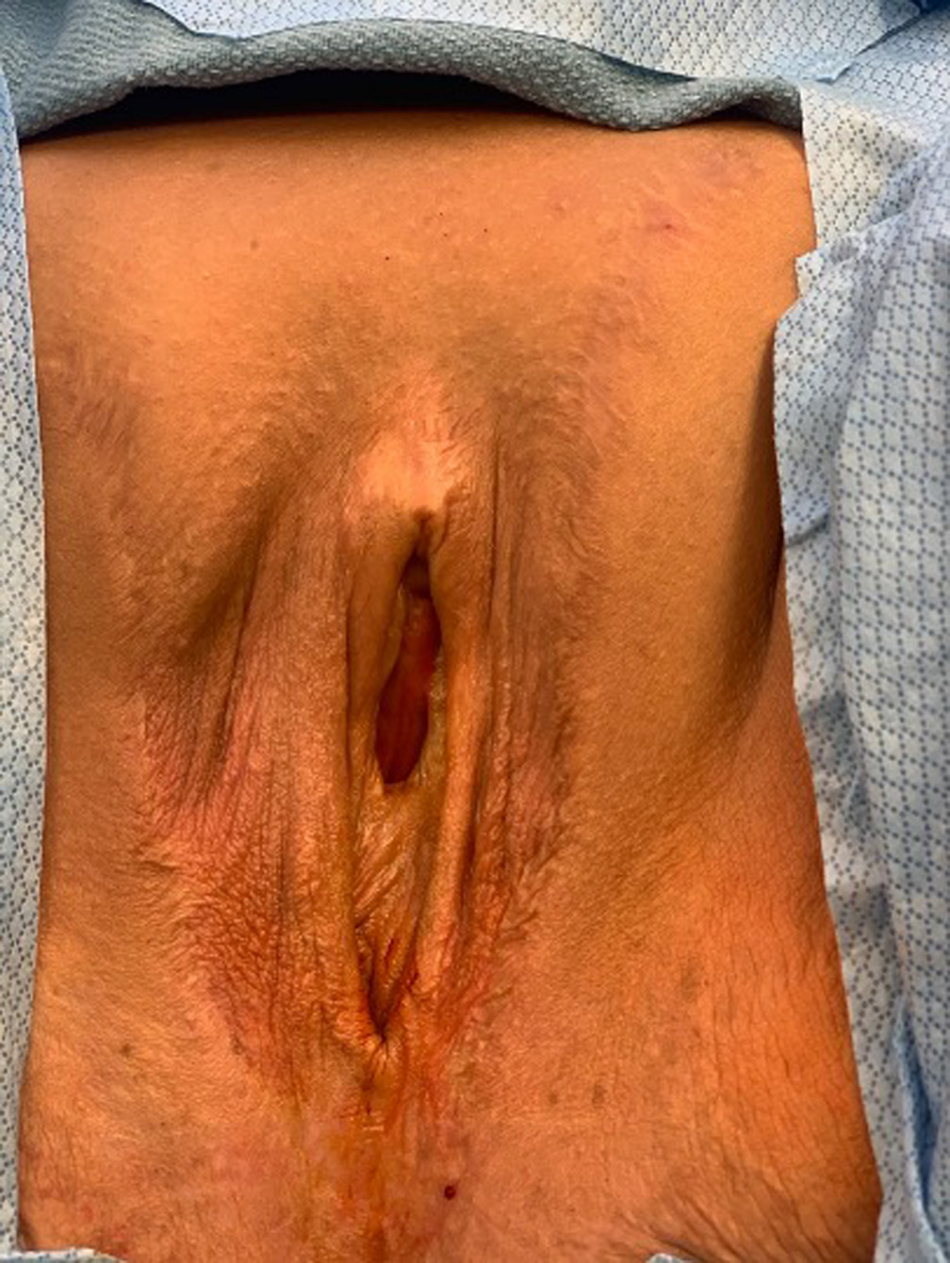
Pre-revision. The right labia majora is retracted. The labia minora are poorly defined and blend in with the medial aspect of the labia majora.

We present a 38-year-old transgender female status post penile inversion vaginoplasty approximately 10 months prior. She initially presented to both urology and plastic surgery clinics with a complaint of asymmetry and poor definition of her labia (Figure 1). In addition, she had bothersome splaying of her urinary stream, which can occur in approximately 9.5% of patients after inversion vaginoplasty and is usually due to meatal stenosis.^14^

Our procedure begins with a cystoscopy to evaluate the urethra and addressing of the urine misdirection and splaying resulting from a lip of tissue on the anterior urethra. No stricture or other pathology was seen in her urethra or bladder on cystoscopy. The lip of tissue deflecting the urine stream was incised and the edges sutured to maintain patency. Next, we lifted an advancement flap for the right labia majora, which had retracted since her initial surgery. At this time, we also dissected medially to create a thin advancement flap for the right labia minora measuring approximately 6 × 2 cm.

**Figure 2.**
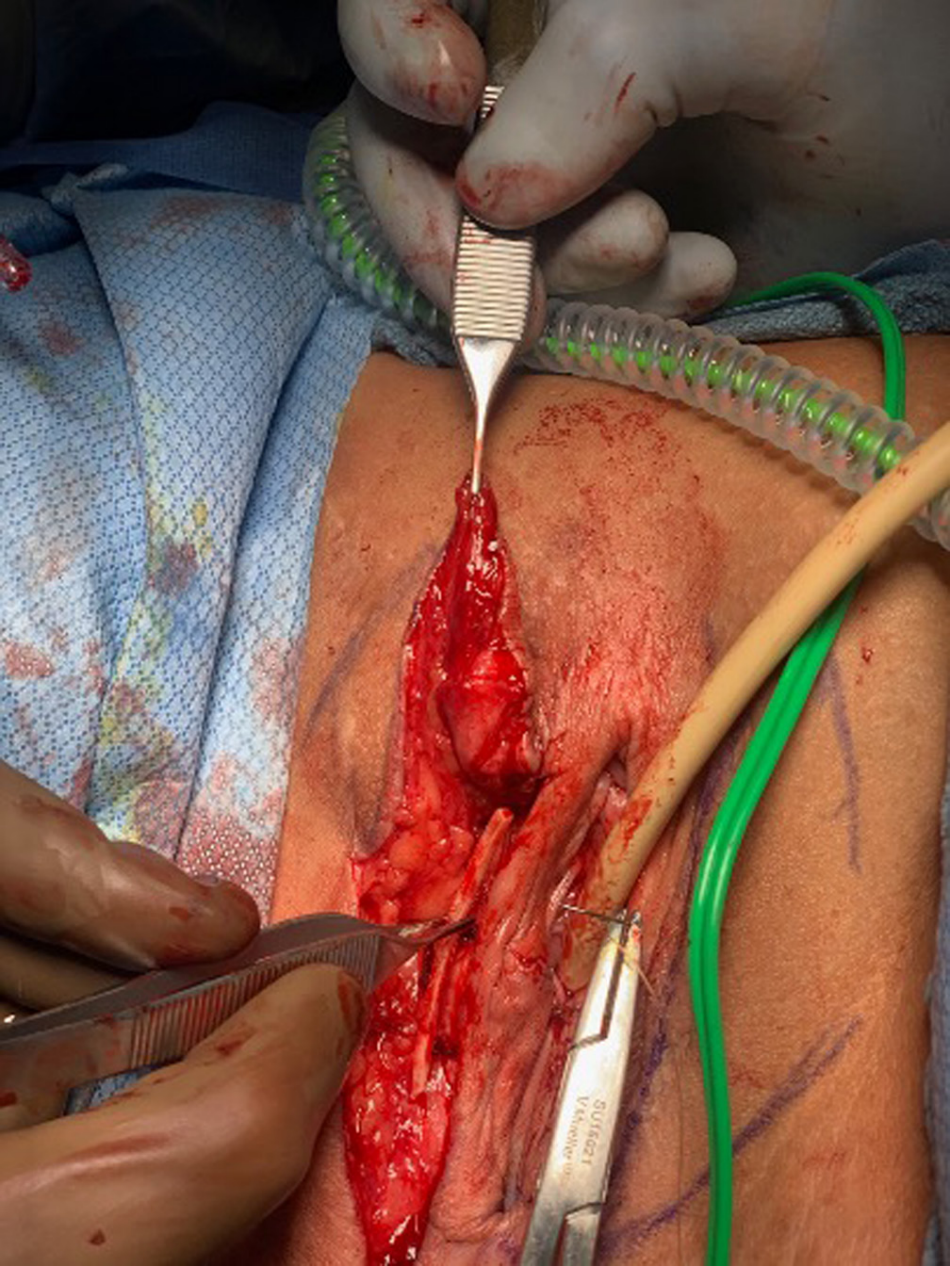
The right labia majora flap has been created and retracted superiorly. The formed fresh frozen cartilage has been shaped and placed in its position with the labia minora flap medially. A Foley catheter had been placed during the urethral revision.

**Figure 3.**
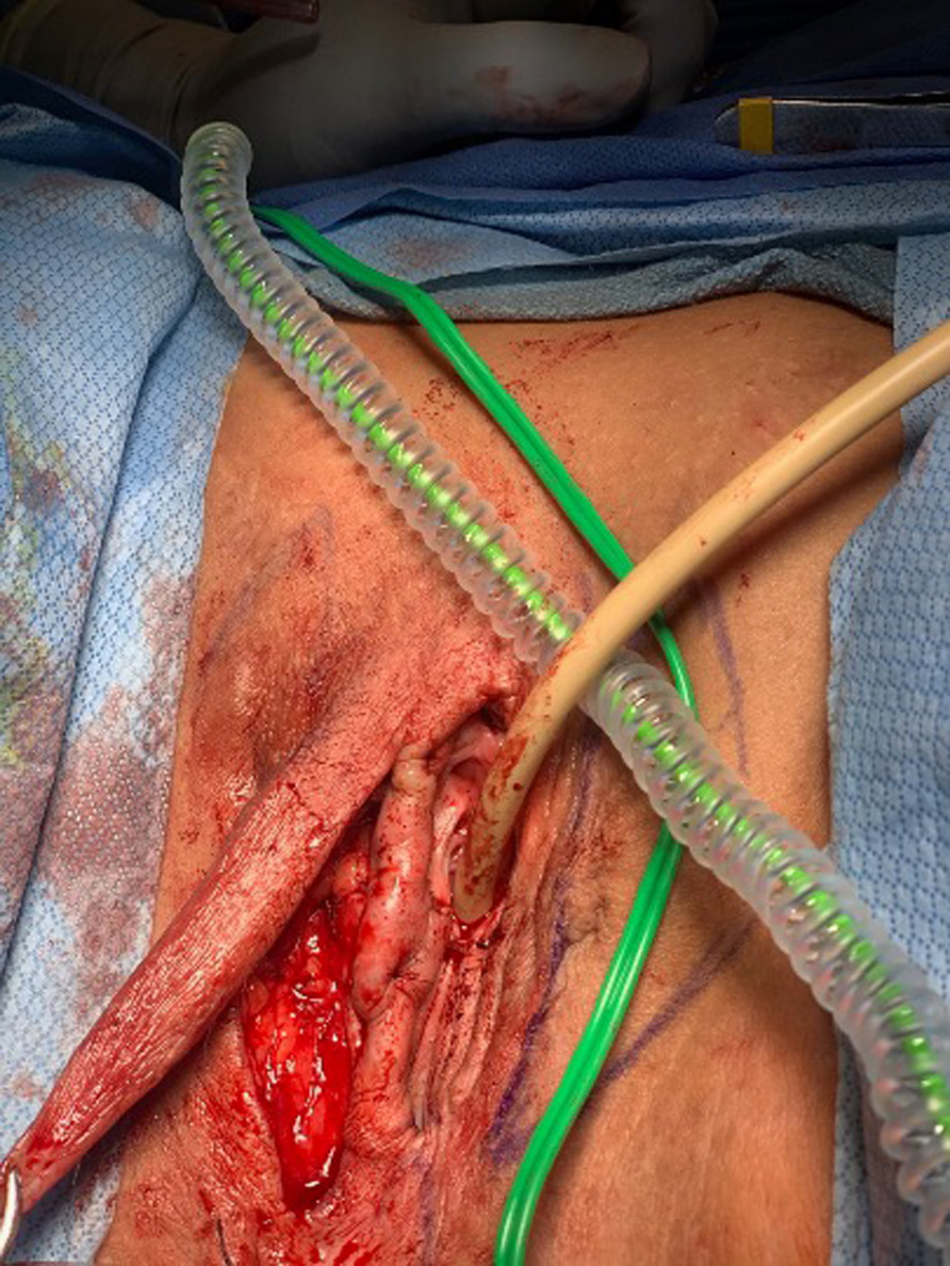
The cartilage has been secured under the right labia minora flap. The right labia majora flap is pulled down to position.

We selected MTF profile cartilage prepared to industry standards and cut to a thin, approximately 4–cm-long section. The cartilage was placed within the previously made labia minora flap before being sutured in place with 4-0 PDS (Figure 2). The labia majora flap was then advanced and inset in a layered fashion using 2-0 PDS for a superficial fascia closure followed by 3-0 Monocryl for interrupted deep dermal and 4-0 Monocryl for final skin approximation (Figure 3). The cartilage provided soft tissue support for the labia minora.

A similar 6 × 2 cm advancement flap was lifted medial to the left labia majora. An identical section of fresh frozen cartilage was cut and sutured in place using 4-0 PDS. The skin was closed with 4-0 Monocryl over the cartilage which again provided definition for the revised labia minora.

**Figure 4.**
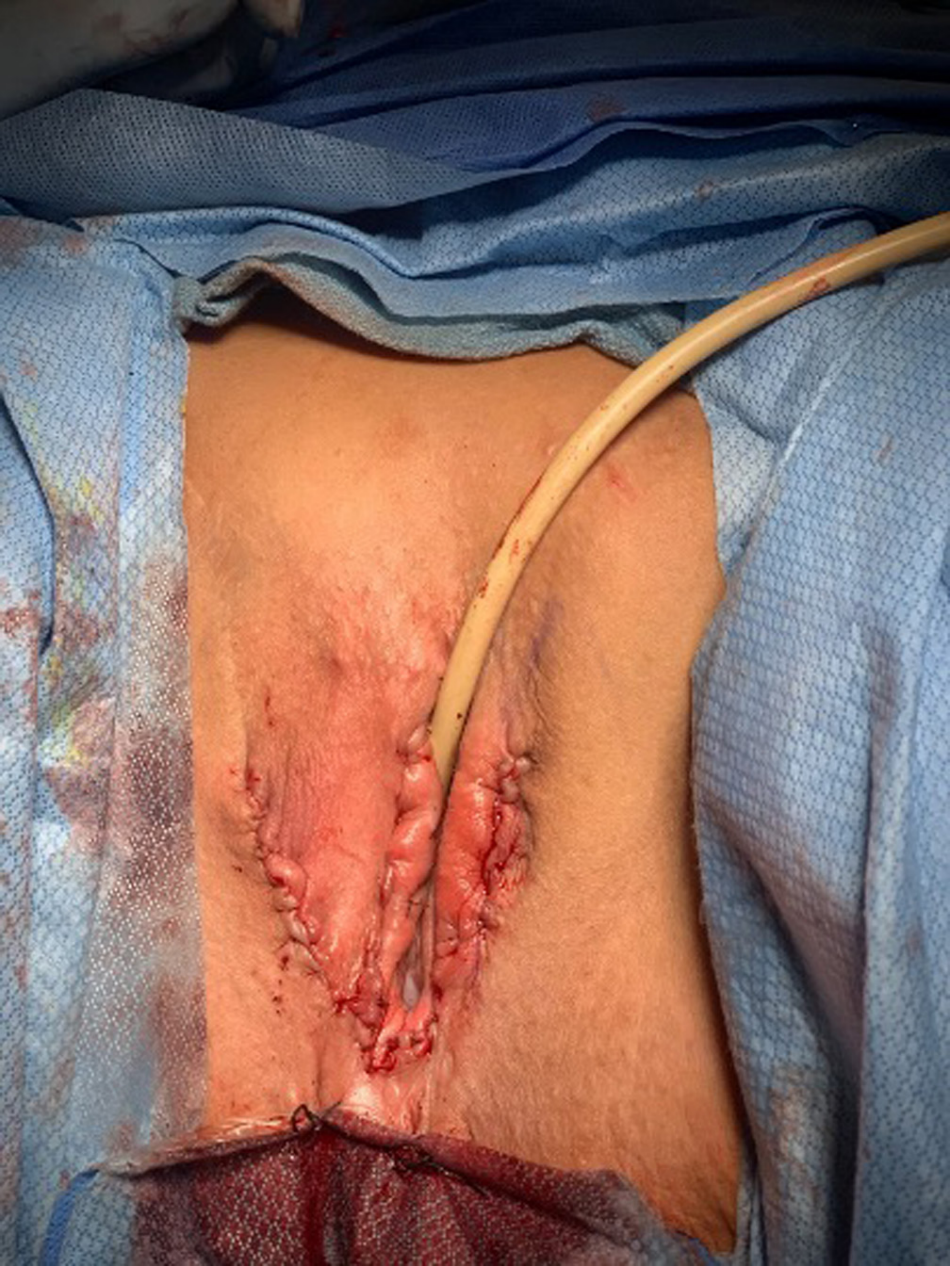
Results at the end of the procedure.

Each labia majora received 15 mL of Reunuva (MTF Biologics, Edison, NJ, USA) to add volume and definition (Figure 4). Another option would have been labial fat grafting, but Renuva was chosen due to its off-the-shelf availability and in an effort to reduce adverse effects.^15^ The patient was safely discharged home post-operatively.

Our institution considers reports of cases including information with less than 3 patients to be exempted by the institutional review board. Our institution does not require research of this type to undergo approval by the ethics committee. The patient was informed that data concerning the case would be submitted for publication, and the patient provided consent.

## Results

**Figure 5.**
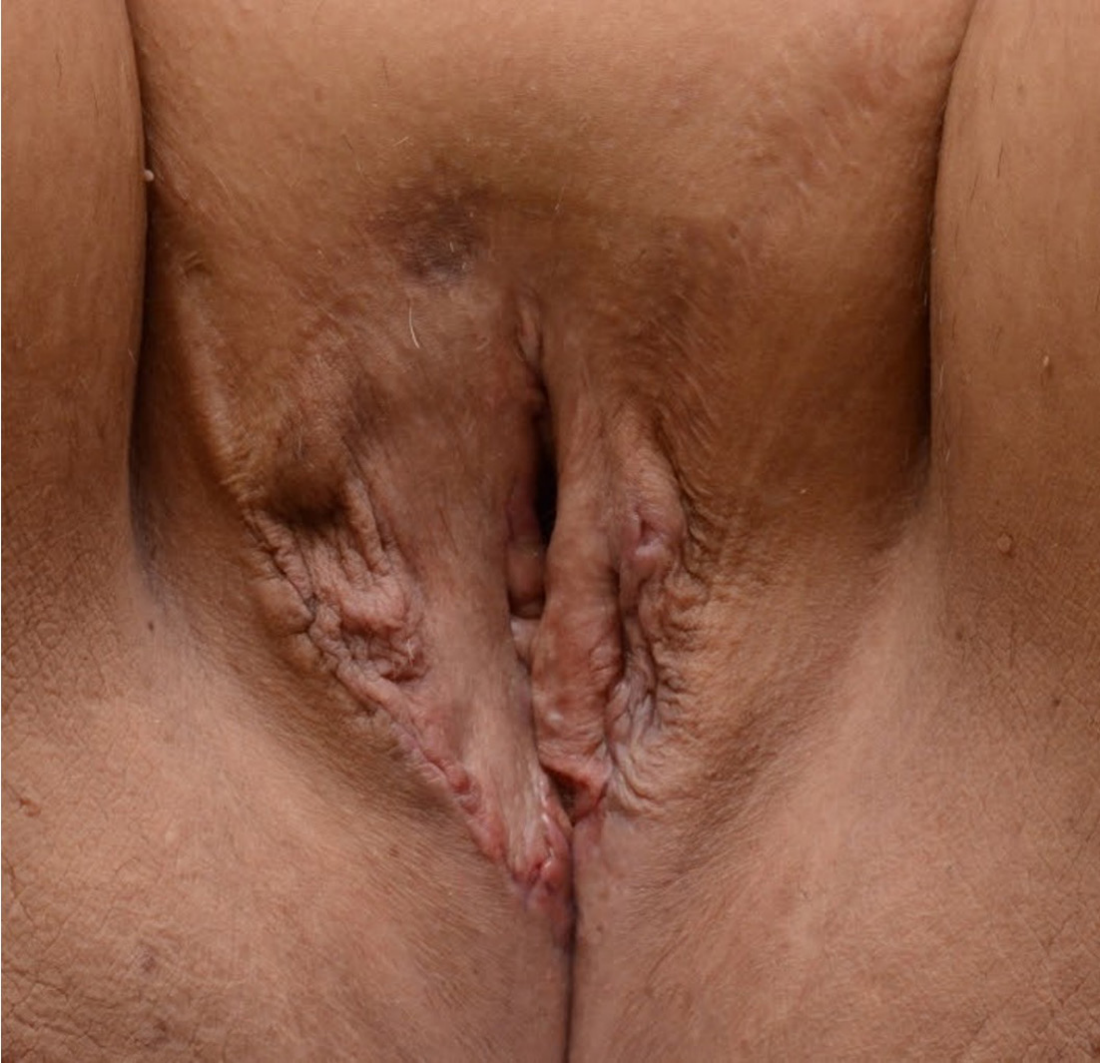
Results 2 weeks post procedure. Asymmetries can be seen. The left labia minora is flipped medially.

Upon follow-up at the 2 weeks mark, she had only slight asymmetries (Figure 5). We believe that the labia will smooth out over the subsequent weeks and provide a more natural appearance. Her urinary misdirection had resolved, and she had no urinary complaints, and thus, a uroflow was not felt necessary. She had no complications post-operatively.

**Figure 6.**
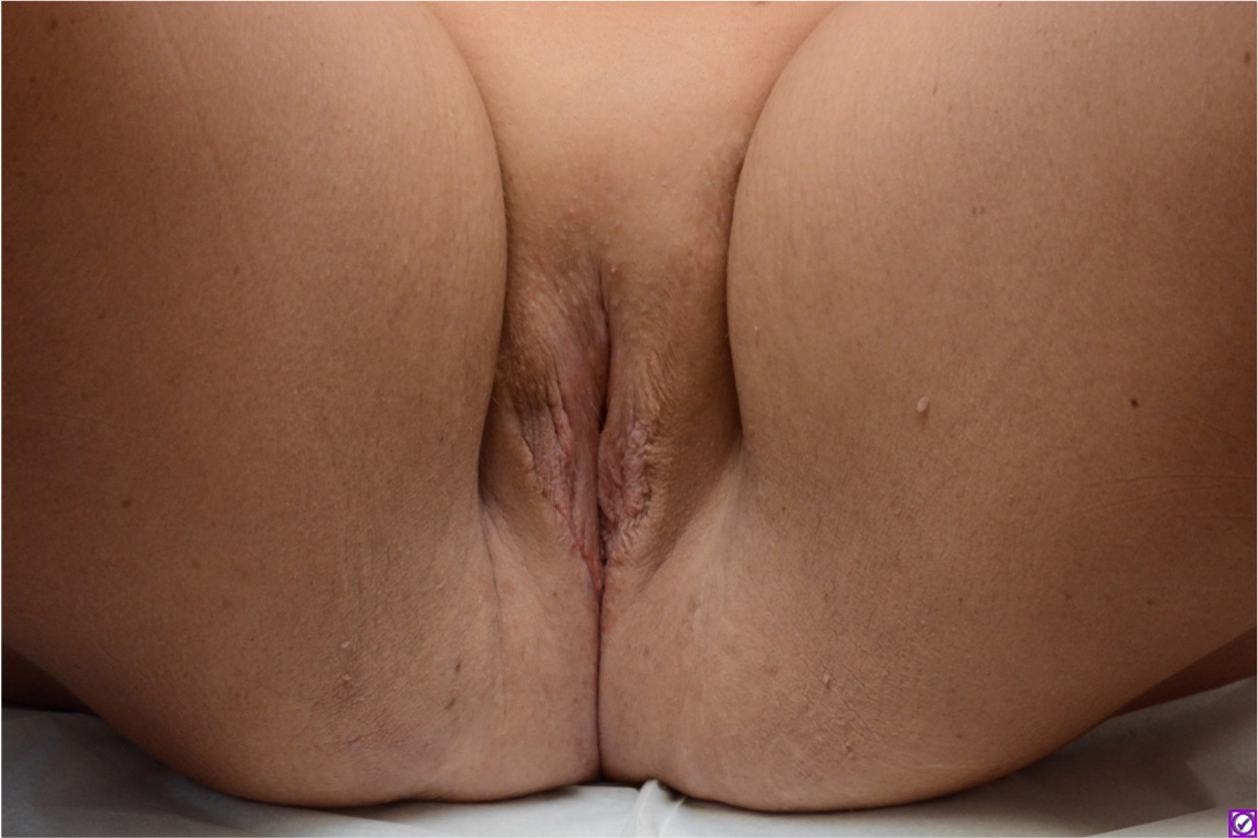
Six weeks post-operatively. Left labia minora is mildly inflected inward. Left labia minora appears slightly retracted superiorly and blended with labia majora.

The patient was followed up 6 weeks post-operatively to assess the result (Figure 6). The left labia minora appears more prominent and with a slight inflection inward. The right labia minora has mildly retracted superiorly and slightly blended with the labia majora. Unfortunately, the patient was unable to follow up after 6 weeks as she did not live local to our institution.

This technique was very effective on the left labia minora; however, there was some loss of definition seen on the right side. This may have been due to the concurrent labia majora advancement flap leading to poor anchoring of the labia minora flap. This technique could be simplified if performed in a patient with already well-defined labia majora.

## Conclusion

This case represents the first known instance within the literature of cartilaginous transplant in the realm of transgender surgery, especially with regard to revision labiaplasty or vaginoplasty. This procedure has the potential to provide additional definition effectively and safely to achieve cosmetic satisfaction; however, additional studies with longer-term follow-up could be useful in optimizing the technique.

The patients urinary splaying was due to a deflecting lip of tissue and not due to stenosis which is more common.^14^ Simply incising the lip and suturing the edges resulted in satisfactory voiding without bothersome misdirection.

The utilization of fresh frozen cartilage allograft for revision labiaplasty provides an exciting and promising addition to the evolving field of gender affirmation. Effective in providing structural as well as aesthetically desirable results elsewhere, cartilage graft can demonstrate similar outcomes when utilized for labiaplasty.
